# Physical Activity Improves Verbal and Spatial Memory in Older Adults with Probable Mild Cognitive Impairment: A 6-Month Randomized Controlled Trial

**DOI:** 10.1155/2013/861893

**Published:** 2013-02-24

**Authors:** Lindsay S. Nagamatsu, Alison Chan, Jennifer C. Davis, B. Lynn Beattie, Peter Graf, Michelle W. Voss, Devika Sharma, Teresa Liu-Ambrose

**Affiliations:** ^1^Department of Psychology, University of British Columbia (UBC), 2136 West Mall, Vancouver, BC, Canada V6T 1Z4; ^2^Department of Physical Therapy, University of British Columbia (UBC), 212-2177 Wesbrook Mall, Vancouver, BC, Canada V6T 1Z3; ^3^Alzheimer Clinic, G37 Purdy Pavilion, UBC Hospital, University of British Columbia (UBC), 2211 Wesbrook Mall, Vancouver, BC, Canada V6T 2B5; ^4^Brain Research Centre, University of British Columbia (UBC), 2211 Wesbrook Mall, Vancouver, BC, Canada V6T 2B5; ^5^Department of Psychology, The University of Iowa, E11 Seashore Hall, Iowa City, IA 52242-1409, USA

## Abstract

We report secondary findings from a randomized controlled trial on the effects of exercise on memory in older adults with probable MCI. We randomized 86 women aged 70–80 years with subjective memory complaints into one of three groups: resistance training, aerobic training, or balance and tone (control). All participants exercised twice per week for six months. We measured verbal memory and learning using the Rey Auditory Verbal Learning Test (RAVLT) and spatial memory using a computerized test, before and after trial completion. We found that the aerobic training group remembered significantly more items in the loss after interference condition of the RAVLT compared with the control group after six months of training. In addition, both experimental groups showed improved spatial memory performance in the most difficult condition where they were required to memorize the spatial location of three items, compared with the control group. Lastly, we found a significant correlation between spatial memory performance and overall physical capacity after intervention in the aerobic training group. Taken together, our results provide support for the prevailing notion that exercise can positively impact cognitive functioning and may represent an effective strategy to improve memory in those who have begun to experience cognitive decline.

## 1. Introduction

Cognitive decline is one of the most pressing health care issues of the 21st century. Currently worldwide, one new case of dementia is detected every seven seconds [1] and the number of people affected is projected to be over 80 million by 2040 [1]. Thus, the societal value of developing effective intervention strategies cannot be overstated [2]. To date, pharmacological interventions for dementia have remained medically challenging at best. As a result, there has been growing interest in exercise training as an alternative intervention strategy. 

The primary aim of our current study was to investigate the efficacy of exercise as an intervention strategy to improve memory performance in older adults who have already begun to experience cognitive decline—namely, those with mild cognitive impairment (MCI). MCI is characterized by cognitive decline that is greater than expected for an individual's age and education level, but does not significantly interfere with everyday function (i.e., instrumental activities of daily living) [3]. Importantly, MCI is a well-recognized risk factor for dementia; longitudinal studies report that seniors with MCI develop Alzheimer's disease at a rate of 10–30% annually [4, 5], compared to 1-2% of seniors without MCI [5]. Thus, MCI represents a critical window of opportunity to intervene and alter the trajectory of both cognitive and functional decline in seniors.

Exercise is a promising strategy for improving cognitive functions. Previous research has found that both resistance training [6, 7] and aerobic training [8] positively impact cognitive functioning and result in functional plasticity in healthy older adults. Furthermore, emerging evidence also suggests that exercise training has cognitive benefits for seniors with MCI. For example, a 24-week home-based physical activity program improved performance on the Alzheimer Disease Assessment Scale-Cognitive Subscale in seniors with probable MCI [9]. Additionally, a six-month aerobic training program improved selective attention and conflict resolution, processing speed, and verbal fluency in senior women with amnestic MCI [10].

 Expanding upon the existing body of knowledge on exercise and cognitive functions, we found that twice-weekly progressive resistance training in our six-month intervention improved associative memory—or the memorization of two items in conjunction—in senior women with probable MCI [11]. These findings were further corroborated by our neuroimaging results, where resistance training was associated with increased activation over time in key cortical regions that subserve associative memory. Importantly, other research groups have found complementary evidence for a relationship between physical activity and memory in seniors with MCI [12]. However, a comparison has not been conducted between resistance and aerobic training for their propensity to improve various forms of memory in older adults who are showing signs of memory decline.

 For our study reported here, we analyzed the secondary outcome measures of our randomized controlled trial that was previously published [11]. Our current analysis was primarily designed to examine the efficacy of both resistance training and aerobic training to improve memory performance in senior women with probable MCI. To this end, we examined the impact of exercise on two distinct forms of memory: (1) verbal memory and learning and (2) spatial memory. The second aim of our study was to determine whether memory performance at the end of the trial might be associated with physical performance measures. While research on the effects of resistance training on cognitive function has been limited, preliminary evidence does suggest that different forms of exercise (e.g., aerobic versus resistance training) alter distinct cognitive processes [6, 11]. Consistent with this idea, Cassilhas and colleagues [13] recently reported differences in underlying molecular mechanisms between the two types of exercise for how they may improve cognitive function; whereas resistance training appeared to increase levels of serum IFG-1, aerobic training increased levels of brain-derived neurotrophic factors (BDNF) in the hippocampus. Therefore, we hypothesized that both types of exercise training would yield beneficial—although potentially divergent—impacts on memory. 

## 2. Methods

### 2.1. Study Design

We conducted a 26-week, single-blinded, randomized trial of exercise (NCT00958867) with assessments at baseline, mid-point, and trial completion. Details of the trial have been reported elsewhere [11].

### 2.2. Participants

Our study only included women due to sex differences in cognitive response to exercise [14]. From April 2009 to August 2009, we recruited participants using advertisements in local media and a memory clinic. Individuals were screened by a standardized telephone interview and by a 30-minute in-person assessment. Women who lived in Vancouver, Canada, were eligible for study entry if they (1) were aged 70 to 80 years; (2) were living independently in their own home; (3) scored ≥24/30 on the Mini-Mental State Examination (MMSE); (4) scored <26/30 on the Montreal Cognitive Assessment (MoCA) [15]; (5) answered “yes” to the question “do you have any difficulty with your memory?” [9]; (6) scored ≥6/8 on the Lawton and Brody [16] Instrumental Activities of Daily Living; (7) had a visual acuity of at least 20/40, with or without corrective lenses; and (8) obtained their physician's clearance to start a supervised exercise program. We excluded those who (1) had a current medical condition for which exercise is contraindicated; (2) had participated regularly in resistance training or aerobic training in the last six months; (3) had a neurodegenerative disease and/or stroke; (4) had a diagnosed psychiatric condition (e.g., depression); (5) had a diagnosis of dementia of any type; (6) did not speak and understand English fluently; or (7) were on oestrogen replacement therapy.

**Figure 1:**
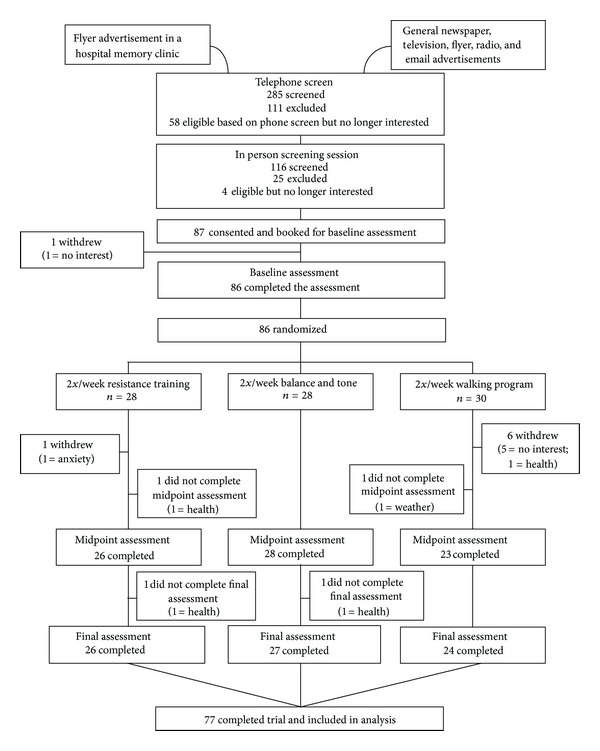
The CONSORT (Consolidated Standards of Reporting Trials) flowchart.

In Figure 1, the CONSORT (Consolidated Standards of Reporting Trial) flowchart shows the number and distribution of participants. Ethical approval was obtained from the Vancouver Coastal Health Research Institute and the University of British Columbia's Clinical Research Ethics Board. All participants provided written informed consent.

### 2.3. Descriptive Variables

Current level of physical activity was determined by the Physical Activities Scale for the Elderly (PASE) self-report questionnaire [17]. The 15-item Geriatric Depression Scale [18] screened for depression. The Functional Comorbidity Index was calculated to estimate the degree of comorbidity associated with physical functioning [19]. We used the Lawton and Brody [16] Instrumental Activities of Daily Living Scale to subjectively assess ability to perform daily activities.

### 2.4. Verbal Memory and Learning

The Rey Auditory Verbal Learning Test (RAVLT) [20] assessed verbal memory and learning. Participants were read a list of 15 common words five times. Immediately after each time, they were required to recall as many words as possible. After the fifth trial, an interference list was presented, after which participants had to spontaneously recall the original words. Finally, participants were required to spontaneously recall the original words after a 20 minute delay. Scores were calculated as the total number of words recalled (1) across the five trials (total acquisition); (2) after the interference list (recall after interference); (3) on the fifth trial minus after the interference (loss after interference); and (4) after the delay (long delay free recall).

### 2.5. Spatial Memory

Spatial memory was assessed using a computerized task developed in-house by one of our co-authors along with her collaborators. This task was chosen because it has previously been found to modulate with physical activity [21–23] and allows for the collection of reaction times and accuracy—rather than just working memory span which other spatial memory tasks provide. Our spatial memory task required participants to recall the spatial location of dots presented on a screen. Specifically, one, two, or three dots appeared at randomly selected locations on the screen for 500 ms. Next, a fixation-cross appeared for 3 s. At the end of the delay, a single red test dot was presented on the screen, either at the same location as one of the previous black dots (match), or at a new location (nonmatch). Subjects were required to indicate whether the red test dot was a match or a nonmatch to any of the previously presented black dots by pressing the designated key on a computer keyboard (“x” = nonmatch; “m” = match). Participants were instructed to respond as quickly and accurately as possible. The entire task consisted of 120 trials (40 trials for each set size, divided into 20 match and 20 nonmatch conditions). Participants were provided with practice trials prior to beginning the test to ensure they understood the task instructions. Reaction times and accuracy were recorded.

### 2.6. Choice Reaction Time

Choice reaction times were collected to use as a covariate in our statistical analyses for computerized tasks measuring reaction times to account for differences in basic processing speed secondary to memory performance [24]. Participants were required to indicate whether a number (1, 2, 3, 4, 6, 7, 8, 9) presented on a computer screen was higher or lower than the number “5”. Numbers were presented individually for 1500 ms in the centre of the screen, and the same number did not repeat twice in a row. Using one hand, they were required to press one button with their index finger if the correct answer was “higher” than five and another button with their middle finger if the number was “lower” than five. Participants were instructed to respond as quickly and accurately as possible.

### 2.7. Physical Performance

General balance and mobility was assessed using the Short Physical Performance Battery, which is a composite score of the following tasks: (1) tandem standing; (2) four-metre walk (gait speed); and (3) chair stands. General cardiovascular capacity was assessed using the Six-Minute Walk Test, where the total distance walked at participants' usual pace in six minutes was measured in metres.

### 2.8. Randomization

The randomization sequence was generated by (http://www.randomization.com) and was concealed until interventions were assigned. This sequence was held independently and remotely by the Research Coordinator. Participants were enrolled and randomized by the Research Coordinator to the twice-weekly exercise groups: resistance training (RT), aerobic training (AT), or balance and tone (BAT). 

### 2.9. Exercise Intervention

The exercise protocol has been reported elsewhere [11]. Briefly, classes began one month after baseline assessments and were held at a fully equipped gym in a research centre. Classes were led by certified fitness instructors who received additional training from the study investigators. The classes were 60 minutes in duration (10-minute warm-up, 40 minutes of core content, and 10-minute cool-down). Attendance was recorded daily, which was used to calculate compliance (i.e., percentage of total classes attended). Strategies were implemented to promote participant engagement [6, 25]. 

#### 2.9.1. Resistance Training

For the RT program, both a Keiser Pressurized Air system and free weights were used [6]. The Keiser-based exercises consisted of biceps curls, triceps extension, seated row, latissimus dorsi pull downs, leg press, hamstring curls, and calf raises. The intensity of the training stimulus was at a work range of six to eight repetitions (two sets). The training stimulus was subsequently increased using the 7RM method—when two sets of six to eight repetitions were completed with proper form and without discomfort. Other key strength exercises included minisquats, minilunges, and lunge walks. 

#### 2.9.2. Aerobic Training

The AT program was an outdoor walking program. The intensity of the training stimulus was at approximately 40% of one's age specific target heart rate (i.e., heart rate reserve; HRR) and progressed over the first 12 weeks to the range of 70% to 80% of HRR. Exercise intensity was monitored through heart rate monitors. Participants also monitored the intensity of their workouts by the Borg's Rating of Perceived Exertion [26] and the “talk” test [27, 28]. 

#### 2.9.3. Balance and Tone

The BAT program consisted of stretching exercises, range of motion exercises, balance exercises, functional sand relaxation techniques [6]. Other than bodyweight, no additional loading (e.g., hand weights, etc.) was applied. This group served to control for confounding variables such as physical training received by traveling to the training centres, social interaction, and changes in lifestyle secondary to study participation.

### 2.10. Adverse Effects

Participants were questioned about the presence of any adverse effects, such as musculoskeletal pain or discomfort, at each exercise session. Instructors monitored participants for symptoms of angina and shortness of breath during the exercise classes.

### 2.11. Statistical Analysis

All analyses were “full analysis set” [29] (defined as the analysis set which is as complete and as close as possible to the intention-to-treat ideal of including all randomized participants). Data were analyzed using IBM SPSS STATISTICS (Version 20).

Performance on the RAVLT was measured using univariate ANOVAs for each outcome measure, with two planned simple contrasts to assess differences between (1) RT versus BAT and (2) AT versus BAT. Baseline scores were entered as covariates. For the spatial memory task, repeated measures ANOVAs were performed to examine changes over the course of the trial in both reaction time and accuracy, with number of items (one, two, or three) as the within-subjects factor and group as the between-subjects factor. The reaction time analysis included choice reaction time as a covariate to account for differences in processing speed [24]. Bivariate Pearson correlations were calculated to examine the relationship between memory and physical performance at trial completion within each group. For all analyses the overall alpha was set at *P* ≤ 0.05. 

## 3. Results

### 3.1. Descriptive Variables, Physical Activity, and Participants

In this trial, 86 participants were recruited and randomized (Figure 1). Baseline demographic and characteristics of the 86 participants are shown in Table 1. Physical activity levels (PASE scores) did not differ significantly between the groups at midpoint (*P* = 0.93) or trial completion (*P* = 0.67). Of the 86 participants, 77 completed the 26-week trial. The number of dropouts was the greatest in the AT group (Figure 1). 

### 3.2. Verbal Memory and Learning

**Figure 2:**
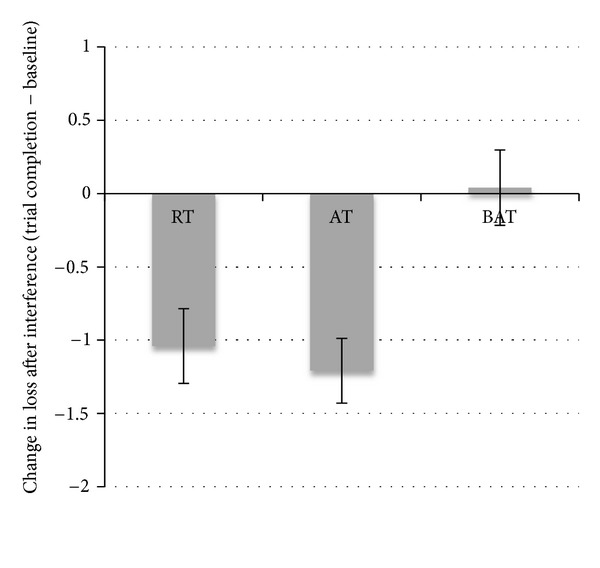
Change between baseline and trial completion in loss after interference on the RAVLT as a function of exercise group. The AT group showed significantly more change compared to the BAT group. Error bars represent standard error of the mean.

Table 2 shows the baseline, mid-point, and trial completion results for verbal memory and learning performance. For the RAVLT, there were no significant between-group differences at trial completion in total acquisition, recall after interference, and long delay free recall (all *P*'s > 0.15). However, there was a significant difference in loss after interference at trial completion between the AT and BAT groups, *P* = 0.04 (Figure 2). Conversely, the RT and BAT contrast for loss after interference was nonsignificant, *P* = 0.20. Overall, loss after interference was reduced by 43.4% and 32.5% in the AT group and the RT group, respectively. In contrast, the BAT group demonstrated a 1.45% increase in loss after interference. The improvement observed in the AT group was not present at mid-point, *P* = 0.71.

### 3.3. Spatial Memory

**Figure 3:**
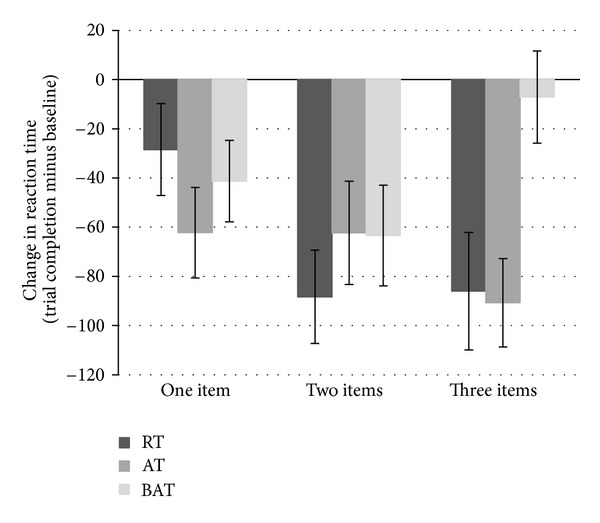
Change between baseline and trial completion on the spatial memory task as a function of number of items presented and exercise group. Both the RT and AT groups showed improved performance compared to the BAT group for the memorization of three items. Error bars represent standard error of the mean.

Table 3 shows the baseline, mid-point, and trial completion results for reaction time and accuracy on the spatial memory task. The exercise groups differed in reaction time changes after completion of the trial as a function of number of items to be remembered. This was evidenced by a significant item by group interaction, *F*(4,100) = 2.41, *P* = 0.05. Examining the plots (Figure 3), the RT and AT groups appear to have improved their reaction times for memorizing the spatial location of three items more than the BAT group. Notably, this between-groups difference was not present at mid-point, *P* = 0.18. There were no significant differences between the exercise groups in accuracy for spatial memory for either mid-point or trial completion (*P* = 0.83 and 0.14, resp.).

### 3.4. Correlations between Memory and Physical Performance

**Figure 4:**
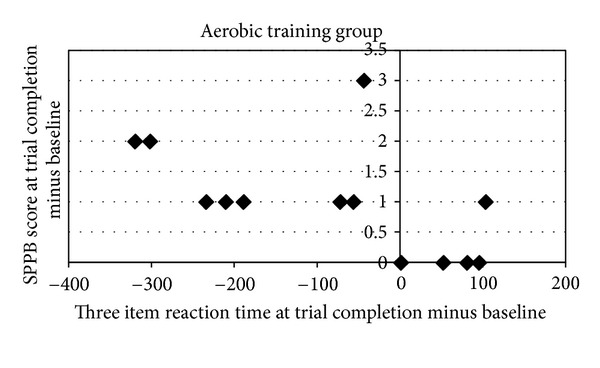
Significant correlations between change in reaction time performance on the spatial memory task and change in performance on the SPPB for the AT group. SPPB performance was negatively correlated with spatial memory reaction time, where better balance and mobility were associated with faster reaction times.

Changes in physical performance as a function of exercise group have been previously reported [11]. In this study, faster reaction times at trial completion compared to baseline during the three-item condition on the spatial memory task were associated with better performance on the SPPB in the AT group (Figure 4). This was confirmed via a significant negative correlation between the two variables, *r*(13) = −0.57,  *P* = 0.04. Spatial memory reaction times and SPPB were not significantly correlated in the RT or BAT groups (*P* = 0.20 and 0.79, resp.). Furthermore, reaction times were not associated with performance on the Six-Minute Walk Test (all *P*'s > 0.17). There were no significant correlations between RAVLT performance and either of the physical performance measures (all *P*'s > 0.37).

### 3.5. Adverse Events

Adverse effects included episodes of shortness of breath that resolved with rest (*n* = 2) and noninjurous falls (*n* = 4). Results of the Chi Square test indicated no significant between-group differences (*P* = 0.54) in the proportion of participants reporting adverse events. 

## 4. Discussion

We analyzed our secondary data from a six-month intervention to examine the effects of aerobic training and resistance training on two distinct forms of memory. Our specific aims were to evaluate whether either type of exercise would improve verbal memory and learning and/or spatial memory and to determine whether an association might exist between postintervention memory performance and physical measures. In this regard, we report three key findings. First, we found that twice-weekly aerobic training for six months remembered significantly more items in the loss after interference condition on the verbal memory test. Second, our results suggest that both types of exercise improved reaction times during the spatial memory test compared to the control group. Last, spatial memory performance appears to be positively associated with physical performance in the aerobic training group after the intervention. The results of our present study extend those from our previous work [11], where we found that resistance training significantly improved associative memory. Within this context, several noteworthy points of discussion follow.

 To begin with, our finding that aerobic exercise significantly improved verbal memory and learning is consistent with previous reports. Specifically, Pereira and colleagues [30] found that three months of aerobic exercise improved performance on the RAVLT. While the benefits of aerobic activity on verbal memory and learning in our study were only observed after six months—compared to three months in the study by Pereira et al. [30], differences in study design may account for this apparent discrepancy. For example, participants in the study by Pereira et al. [30] were young, healthy adults who engaged in aerobic activity four times per week; this is contrasted with older adults in our study who were already experiencing cognitive decline and exercised twice per week. This suggests that a higher dose of exercise may result in observable changes in memory more quickly. It is worth mentioning that in our study, the resistance training group also showed a greater reduction in loss after interference after the trial compared with the control group, although this change was not significant. Nevertheless, future studies with larger sample sizes may discover that resistance training does yield similar benefits to aerobic training for verbal memory performance. 

 Second, we found that both of our experimental exercise groups showed improved reaction time performance for recalling the spatial location of three items, as compared to the balance and tone group. Task performance on the spatial memory test has been shown to systematically decline as a function of load [31]. That the between-group difference was solely observed for three items—the most difficult condition for the spatial memory task—suggests that exercise distinctively improves higher-level cognitive processing required for more complicated tasks. These findings directly support those from previous studies, where both resistance and aerobic training improved executive functioning—such as selective attention and conflict resolution, as measured by the Stroop task [6, 8, 11]. 

 Third, in light of our initial findings regarding improved associative memory performance after six months of twice-weekly resistance training, the results of our present study suggest that different types of exercise may selectively target distinct cognitive processes—and their underlying neural correlates. To recapitulate, we previously reported that resistance training resulted in improved associative memory performance and increased functional activation in three key regions of the cortex: the right lingual and occipital-fusiform gyri and the right frontal pole [11]. In contrast, here we found that *both* types of exercise training led to improved spatial memory. Importantly, spatial memory has neural underpinnings in the hippocampus [21, 32], thus suggesting that both forms of exercise training may impact hippocampal structure and/or function. Indeed, it has been established that the hippocampus is the structure most sensitive to exercise-induced change via neurogenesis and cell proliferation. For example, aerobic exercise has been found to increase hippocampal volume and levels of BDNF—a neurotrophic factor involved in cell growth and survival and memory promotion [21]. Thus, while there are multiple potential mechanisms to account for the relationship between cognitive functions and physical activity, such as increased cerebral blood flow [33], reduced neuroinflammation [34], and contribution of white matter hyperintensities [35], we can speculate that underlying changes in hippocampal structure and/or function may be a mediating observed relationship between spatial memory and physical performance.

Finally, the link between physical activity and cognitive functioning is further supported by the significant correlation we have reported between our measure of overall physical performance and spatial memory in the aerobic training group. Notably, these results correspond to our previous findings that improvements in conflict resolution and selective attention, as measured by Stroop performance, were significantly correlated to improved gait speed after 12 months of resistance training [6]. That these two studies found relationships between different types of exercise (aerobic versus resistance training) and two different measures of cognitive function further supports the notion presented above that the two types of exercise may target distinct molecular pathways [13]—and thus, modify different subtypes of cognitive function. However, in combination, evidence from these two studies demonstrate that higher levels of physical performance are associated with better cognitive performance. Given that there are multiple ways to improve general physical performance levels, our results therefore suggest that individuals may gain cognitive benefits from a wide variety of exercise options. Future work is needed to explore this possibility.

The conclusions of our study are tempered by our exclusion of men and those older or younger than 70–80 years old. Additionally, our study was only powered to compare the resistance training versus the control group and the aerobic training versus the control group; therefore, we were unable to directly compare changes in performance between our two exercise groups. Hence, future research on how our results may apply to the broader population with larger sample sizes is warranted. 

In sum, our study provides preliminary evidence that multiple benefits for memory can be observed after six months of exercise training. However, the mechanisms behind how resistance training and aerobic training may differentially impact cognition remain unclear; thus future work should be aimed at further understanding the contribution of each type of exercise to cognitive functioning, functional plasticity, and brain structure. Furthermore, while our study did find performance improvements after six months, we did not see comparable changes after only three months using a twice-weekly exercise protocol. Therefore, the dose-response relationship of exercise needs to be elucidated so that future recommendations for the most effective program can be translated to health care practitioners and the public. 

## Figures and Tables

**Table 1:** 

Variable^a^	BAT (*n* = 28)	AT (*n* = 30)	RT (*n* = 28)	Total *N* = 86
	Mean (SD)	Mean (SD)	Mean (SD)	Mean (SD)
Age, years	75.1 (3.6)	75.6 (3.6)	73.9 (3.4)	74.9 (3.5)
Height, cm	158.2 (7.3)	159.2 (5.9)	158.7 (7.0)	158.7 (6.7)
Weight, kg	66.4 (14.0)	64.8 (12.8)	65.2 (10.7)	65.4 (12.4)
Physical activity scale for the elderly	133.2 (78.1)	121.6 (52.9)	151.8 (74.9)	135.2 (69.52)
Education, No (%)				
Less than grade 9	0 (0)	2 (2.3)	0 (00	2 (2.3)
Grade 9 to 12 without certificate or diploma	5 (5.8)	3 (3.5)	2 (2.3)	10 (11.6)
High school certificate or diploma	7 (8.1)	7 (8.1)	7 (8.1)	21 (24.4)
Trades or professional certificate or diploma	5 (5.8)	2 (2.3)	3 (3.5)	10 (11.6)
University certificate or diploma	8 (9.3)	8 (9.3)	8 (9.3)	24 (27.9)
University degree	3 (3.5)	8 (9.3)	8 (9.3)	19 (22.1)
Geriatric depression scale^b^	1.0 (1.8)	1.1 (1.8)	1.4 (2.0)	1.2 (1.8)
Functional comorbidities index^c^	2.6 (2.2)	2.9 (1.5)	3.0 (1.9)	2.8 (1.8)
Instrumental activities of daily living^d^	7.8 (0.5)	7.8 (0.5)	7.7 (0.8)	7.8 (0.6)
Montreal cognitive assessment^e^	22.5 (2.8)	22.2 (2.8)	21.4 (3.4)	22.1 (3.0)
Minimental state examination^f^	27.1 (1.7)	27.4 (1.5)	27.0 (1.8)	27.2 (1.6)
Exercise class compliance, %	59 (14.8)	60 (18.7)	54 (14.7)	57 (16.1)

Abbreviations: BAT: balance and tone; AT: aerobic training; RT: resistance training.

^a^Unless otherwise indicated, data are expressed as mean (SD). Percentages (%) have been rounded and may not total 100.

^b^Maximum was 15 points.

^c^Maximum was 18 points.

^d^Maximum was 8 points.

^e^Maximum was 30 points.

^f^Maximum was 30 points.

**Table 2:** Mean values (SDs) for RAVLT performance.

Variable	Baseline	Midpoint	Final
	Mean (SD)	Mean (SD)	Mean (SD)
RT	*n* = 25	*n* = 24	*n* = 25

Total acquisition	40.88 (8.36)	46.79 (12.68)	44.36 (11.29)
Recall after interference	7.48 (2.50)	8.58 (3.89)	8.52 (3.50)
Loss after interference	3.2 (1.85)	3.38 (2.63)	2.16 (1.80)
Long delay free recall	7.52 (2.76)	8.83 (4.10)	7.79 (4.23)

AT	*n* = 24	*n* = 23	*n* = 24

Total acquisition	39.58 (9.04)	47.22 (11.01)	43.38 (10.95)
Recall after interference	7.83 (3.21)	8.83 (3.73)	9.29 (3.21)
Loss after interference	2.79 (1.79)	2.87 (2.32)	1.58 (1.86)
Long delay free recall	7.46 (3.08)	8.91 (4.28)	8.13 (3.70)

BAT	*n* = 25	*n* = 24	*n* = 25

Total acquisition	41.00 (8.63)	43.75 (9.55)	43.00 (9.61)
Recall after interference	7.60 (2.81)	8.54 (3.22)	8.12 (3.52)
Loss after interference	2.76 (1.92)	2.71 (2.05)	2.80 (2.58)
Long delay free recall	7.84 (3.33)	8.26 (3.68)	8.04 (3.36)

Abbreviations: BAT: balance and tone; AT: aerobic training; RT: resistance training.

**Table 3:** Mean values (SDs) for spatial memory performance.

Variable	Baseline	Midpoint	Final
	Mean (SD)	Mean (SD)	Mean (SD)
RT	*n* = 19	*n* = 18	*n* = 19

Reaction time, ms			
One item	981.02 (141.18)	941.05 (167.40)	952.57 (193.75)
Two items	1036.40 (176.36)	997.00 (196.59)	948.09 (219.19)
Three items	1092.71 (148.86)	1072.64 (189.75)	1006.69 (189.75)
Accuracy^a^			
One item	0.76 (0.17)	0.82 (0.14)	0.74 (0.17)
Two items	0.81 (0.16)	0.81 (0.13)	0.75 (0.19)
Three items	0.72 (0.15)	0.78 (0.13)	0.73 (0.17)
Choice reaction time, ms	895.26 (158.07)	880.42 (149.10)	889.41 (163.19)

AT	*n* = 17	*n* = 16	*n* = 17

Reaction time, ms			
One item	958.77 (161.91)	927.12 (179.21)	896.51 (172.92)
Two items	1013.56 (198.95)	982.32 (146.48)	951.22 (165.06)
Three items	1093.65 (186.34)	1039.24 (172.98)	1002.91 (195.64)
Accuracy			
One item	0.73 (0.11)	0.79 (0.13)	0.77 (0.12)
Two items	0.75 (0.15)	0.84 (0.10)	0.84 (0.09)
Three items	0.71 (0.11)	0.78 (0.10)	0.74 (0.09)
Choice reaction time, ms	877.82 (128.62)	894.49 (130.15)	910.08 (164.62)

BAT	*n* = 19	*n* = 19	*n* = 19

Reaction time, ms			
One item	993.26 (155.19)	968.55 (179.74)	952.00 (178.40)
Two items	1070.66 (210.78)	1010.36 (198.28)	1007.27 (187.55)
Three items	1084.62 (215.32)	1061.94 (190.76)	1077.48 (223.07)
Accuracy			
One item	0.73 (0.14)	0.74 (0.14)	0.79 (0.12)
Two items	0.76 (0.13)	0.76 (0.15)	0.79 (0.12)
Three items	0.71 (0.14)	0.68 (0.15)	0.74 (0.10)
Choice reaction time, ms	864.91 (111.95)	885.06 (169.32)	838.30 (101.36)

Abbreviations: BAT: balance and tone; AT: aerobic training; RT: resistance training.

^a^Maximum was 1.00.
